# Contribution of Short-Time Occlusion of the Amblyopic Eye to a Passive Dichoptic Video Treatment for Amblyopia beyond the Critical Period

**DOI:** 10.1155/2019/6208414

**Published:** 2019-08-28

**Authors:** Lauren Sauvan, Natacha Stolowy, Danièle Denis, Frédéric Matonti, Frédéric Chavane, Robert F. Hess, Alexandre Reynaud

**Affiliations:** ^1^Department of Ophthalmology, CHU NORD, Marseille, France; ^2^Institut de Neurosciences de la Timone (INT), Centre National de la Recherche Scientifique (CNRS) and Aix-Marseille Université (AMU), Marseille, France; ^3^Centre Paradis Monticelli, Marseille, France; ^4^McGill Vision Research, Department of Ophthalmology and Visual Sciences, McGill University, Montreal, Quebec, Canada

## Abstract

Dichoptic movie viewing has been shown to significantly improve visual acuity in amblyopia in children. Moreover, short-term occlusion of the amblyopic eye can transiently increase its contribution to binocular fusion in adults. In this study, we first asked whether dichoptic movie viewing could improve the visual function of amblyopic subjects beyond the critical period. Secondly, we tested if this effect could be enhanced by short-term monocular occlusion of the amblyopic eye. 17 subjects presenting stable functional amblyopia participated in this study. 10 subjects followed 6 sessions of 1.5 hour of dichoptic movie viewing (nonpatched group), and 7 subjects, prior to each of these sessions, had to wear an occluding patch over the amblyopic eye for two hours (patched group). Best-corrected visual acuity, monocular contrast sensitivity, interocular balance, and stereoacuity were measured before and after the training. For the nonpatched group, mean amblyopic eye visual acuity significantly improved from 0.54 to 0.46 logMAR (*p* < 0.05). For the patched group, mean amblyopic eye visual acuity significantly improved from 0.62 to 0.43 logMAR (*p* < 0.05). Stereoacuity improved significantly when the data of both groups were combined. No significant improvement was observed for the other visual functions tested. Our training procedure combines modern video technologies and recent fundamental findings in human plasticity: (i) long-term plasticity induced by dichoptic movie viewing and (ii) short-term adaptation induced by temporary monocular occlusion. This passive dichoptic movie training approach is shown to significantly improve visual acuity of subjects beyond the critical period. The addition of a short-term monocular occlusion to the dichoptic training shows promising trends but was not significant for the sample size used here. The passive movie approach combined with interocular contrast balancing even over such a short period as 2 weeks has potential as a clinical therapy to treat amblyopia in older children and adults.

## 1. Introduction

Amblyopia is a neurodevelopmental disorder arising from abnormal visual experience during childhood over a period called “the period of susceptibility” or “the critical period” [1–5]. It mainly manifests itself by a loss of binocular function, reduced visual acuity in one eye, the amblyopic eye, and it is the most frequent cause of unilateral visual loss in childhood. Its prevalence is around 1-3% of the general population [6–12]. The presently accepted treatment for amblyopia consists of full optical correction [13] and monocular patching of the nonamblyopic eye to force the use of the amblyopic eye [14]. This treatment is only successful for young children, and it has been assumed that older children and particularly adults lack sufficient brain plasticity. Thus, no treatment is offered to these older patients as their amblyopia is thought to be fixed [1–5, 15, 16].

It is now well established that amblyopia is associated with cortical dysfunction at monocular and binocular sites [17–21]. One binocular theory suggested that it is the consequence of an excess of interocular suppression [22–24]. Therefore, binocular training strategies have emerged, focusing on treating the primary binocular disorder [25–37]. These are based on dichoptic image presentation that the subject needs to binocularly combine to have full information content of a global motion stimulus [24], a video game [27, 28, 38], a movie [36, 39, 40], or an altered reality device [25, 41]. Binocular fusion only occurs if the contrast of the image seen by the nonamblyopic eye is reduced sufficiently to address the interocular imbalance resulting from suppression. At first, such strategies involved an active participation of the subjects playing a dichoptic video game where success depended on using information simultaneously presented to each eye [27, 28, 31, 35, 38, 42–46]. There is also evidence that video game training in general can aid bilateral amblyopia [47].

However, not all patients want to play video games, and some children/adults are so amblyopic that it is not possible to resolve the features necessary to play video games. Hence, more recently, a general application of the same contrast balancing principles has been applied to natural scene stimuli with either passive dichoptic movie watching [36, 37] or augmented reality [25]. These procedures are based on the presentation of complementary images in the two eyes. Passive dichoptic movie watching resulted in benefits for visual acuity in children [36, 39] but has not been tested on adults yet. In principle, this method could be applied to the passive viewing of any video content such as sporting programs, movies, or children's animations [40]. Here, we tested this procedure for the first time on amblyopic adults and children with stable and resistant amblyopia which could not be treated with standard procedures.

Binocular training methods are an improvement on the current patching approach because they are better accepted [31, 37] and they aim to obtain a better binocular outcome. They engage binocular viewing and in doing so improve the visual acuity of the amblyopic eye. They are thought to operate by utilizing the residual brain plasticity that remains after the critical period of visual development. Recently, another approach has also demonstrated the residual visual plasticity in normal adults [38, 39]. This involves changes in ocular dominance that occur after just 1-2 hours of monocular occlusion. Interestingly, this short-term monocular occlusion results in a strengthening of the deprived eye which is the opposite of what occurs during the critical period in early life. This shift in dominance is only transient, lasting about 1 hour [48–51]. This has also been shown in adults with amblyopia [52]. The dominance shift for amblyopes is in the same direction as that found for normals, namely, the deprived eye becomes stronger, but it can be of larger magnitude and longer duration. Thus, the binocular imbalance that characterizes amblyopia can be manipulated for a certain duration by occluding the amblyopic eye, the opposite of classical patching therapy. The hypothesis is that the decrease in sensory stimulation during the deprivation induces a contrast gain increment to boost the sensitivity of the patched eye. Since it occurs rapidly, the mechanism underlying this contrast gain increment is suspected to involve a change of the excitatory/inhibitory balance [48–54]. However, this relatively rapid patching effect (few hours) may be quite different to more standard binocular training procedures which operate on a relatively long timescale (weeks). These long-term training procedures may involve a different plasticity mechanism, for example, by establishing new synaptic connections [55, 56].

In this study, we ask whether binocular training based on passive dichoptic movie viewing could, by way of a change in brain plasticity, increase the visual function of subjects with a stable amblyopia who are beyond the normal treatment period for classical patching. Secondly, we wondered if the effects of such a dichoptic treatment protocol could be enhanced by short-term monocular occlusion, specifically carrying out the training during the time window where the occlusion has temporarily rebalanced the excitation-inhibition ratio. To answer this question, we combined these two approaches and asked the subjects to wear an eye patch for two hours prior to undergoing binocular training sessions involving passive dichoptic movie watching.

## 2. Material and Methods

### 2.1. Participants

17 amblyopic subjects were included in our study aged from 9 to 67 yo, mean age 34 yo. The criteria for including subjects in the experiment were the following. Subjects had to present with functional amblyopia, secondary to strabismus or anisometropia or both. Their visual acuity had to be stable for at least one year before inclusion, and children under 12 years old had to go through at least six months of conventional occlusion therapy to make sure that amblyopia was stable and resistant. Best-corrected visual acuity (BCVA) in the amblyopic eye had to be higher or equal to 0.2 logMAR, or BCVA difference between the two eyes had to be at least equal to 0.2 logMAR. The strabismus angle had to be lower or equal to 15 prism diopters. We excluded subjects with organic amblyopia, congenital strabismus, presenting any visual or neurologic disease or presenting developmental delay. For the first examination, each participant had to fill out a questionnaire about his/her medical history, and more specifically on the previous treatments, he/she might have had for amblyopia and the observance of these treatments optical correction, occlusion therapy, and strabismus surgery. Clinical details of the amblyopic subjects are reported in Table 1.

Subjects had to wear their full optical correction for all the testing and training procedures. Five subjects who had anisometropia (S1, S7, S8, S9, and S17) did not wear any optical correction before inclusion. For these subjects, we made them wear their adapted optical correction only during testings and dichoptic movie viewing sessions, which was equivalent in total to approximately 12 hours with an optical correction (see Table 1). This is insufficient itself to explain any improvement in the visual functions in terms of spectacle adaptation [13].

Subjects were allocated into one of two intervention groups: the nonpatched group (10 subjects, see details in Table 1), who only followed the dichoptic movie training (see procedures), and the patched group (7 subjects, details in Table 1), who were subjected to monocular occlusion of the amblyopic eye prior to each training session with the dichoptic movies. Subject allocation to each group was mainly determined by their ability to be patched two hours before coming to the lab.

The study took place in the Ophthalmology Department of La Timone Hospital in Marseille. Written informed consent was obtained from all patients or parents/guardians.

### 2.2. Procedures

Subjects underwent a binocular training procedure. The nonpatched group followed a procedure of six 1.5 hour sessions of dichoptic movie viewing to train their binocular vision similar as in Li et al. [36] (one subject could only undergo 5 sessions, see exact duration per subject in Table 1). The patched group followed the same procedure except that they had to wear an occluding patch over their amblyopic eye for two hours prior to each training session which was removed right before the dichoptic movie viewing. The patch was a standard occlusive adhesive-on-skin Ortopad patch. The patients were shown how to wear the patch in the assessment session. Then, they had to put them on by themselves two hours before they came to the lab for the dichoptic movie viewing session.

A battery of visual function tests detailed below was used to examine the effects of binocular training. It involved monocular visual acuity, contrast sensitivity, interocular balance, and stereosensitivity, tested in that order, each test lasting 5 to 10 minutes. The baseline measures were assessed during a first preliminary assessment session, a few days before the actual beginning of the training. The training outcome measures were realized at the end of the last training session. A follow-up test was also performed approximately one month after the training, but only 10 subjects could come back for this test (see details in Table 1).

### 2.3. Dichoptic Movie Design

**Figure 1:**
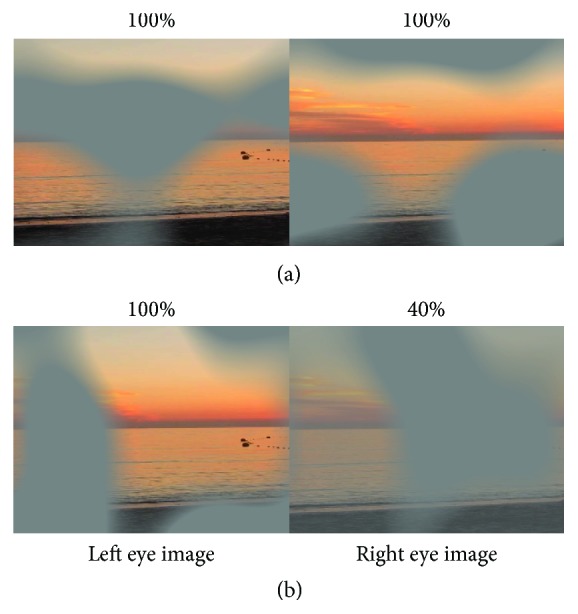
Illustration of the dichoptic movies. The two eyes' views are shown side by side. Complementary patterned image masks composed of irregularly shaped blobs were overlaid over the images seen by the two eyes. The shape and location of the blobs were varied dynamically every 10 seconds. (a) 100% contrast images were presented to the two eyes. (b) A 100% contrast image is presented to the left eye, and an image with a contrast reduced to 40% is presented to the right eye. Movie examples are available as supplementary material. Source video: Lauren Sauvan, wikimedia commons/CC-0.

Patients strengthened their binocular vision by passively watching dichoptic movies. A digital mask composed of irregularly shaped blobs was applied on the images seen by the amblyopic eye, and the inverse mask was applied to the images seen by the fellow eye (Figure 1(a) and example Supplementary [Supplementary-material supplementary-material-1]). Therefore, parts of the image were only seen by one eye and complementary parts were only seen by the other eye [36]. Therefore, to perceive a completed coherent picture, it was necessary to combine information seen by both eyes. The shapes and locations of the masks were varied over time. The contrast of the image seen by the amblyopic eye was fixed to its maximum, and the contrast of the image seen by the fellow eye was based on the results of the binocular balance contrast sensitivity baseline measure (Figure 1(b) and example Supplementary [Supplementary-material supplementary-material-1]). Under these conditions of unequal interocular contrasts, suppression is reduced to the point where information can be combined between the two eyes and the videos perceived stably as a coherent whole. These movies were displayed on a linearized wide passive 3D LG 32LB650V 32^″^ screen, 1920 × 1080 px, 60 Hz (LG Electronics USA; Englewood, NJ) with polarized glasses at a distance of 120 cm, spanning 32° of visual angle.

If the subject perceived the full picture of the movie during a session, then, the contrast in the fellow eye was increased by 10% for the next session. In practice, all subjects always perceived the full picture, and so the contrast was increased in each session. Thus, it happened that it reached the maximal value of 100% for some subjects before the end of the training. Participants confirmed that they could still see the two eye images during each session. This follows the dichoptic balancing principles that have been validated by video games in a number of different studies [26–33, 35–37].

### 2.4. Visual Function Assessment

#### 2.4.1. Visual Acuity

Visual acuity was measured using a logarithmic letter chart in standardized conditions (logarithmic visual acuity chart “2000”).

#### 2.4.2. Contrast Sensitivity Function

**Figure 2:**
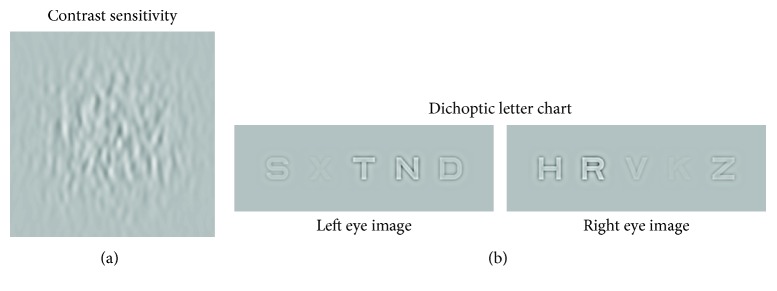
Test stimuli illustrations. (a) qCSF stimulus illustration. In a single-interval identification task, the subject had to judge the orientation (horizontal or vertical) of a filtered noise pattern of varying spatial frequency and contrast. (b) Dichoptic letter chart illustration. Five letters of 2 c/d were presented at various contrasts to the left eye and 5 different letters with complementary contrasts to the right eye at the same spatial locations. So when viewed with both eyes, letters appeared overlapping on screen. Adapted from Kwon et al. [61]; Birch et al. [62].

Monocular contrast sensitivity as a function of spatial frequency was measured using the quick contrast sensitivity function [57]. This is a Bayesian adaptive method which determines the optimal pair of spatial frequency and contrast to test at each trial in order to maximize the information about the contrast sensitivity function. Over the course of 100 trials, the participant had to identify in a single-interval identification task the orientation (horizontal or vertical) of a spatially filtered noise pattern at these set spatial frequencies and contrasts (Figure 2(a)). This method has already been validated on amblyopic subjects [58, 59]. This test was performed on the same equipment as the movie viewing except that participants wore an eyepatch to test monocular vision. Full details of the procedure are given in Reynaud et al. [60].

#### 2.4.3. Interocular Balance

Interocular balance was measured with the dichoptic letter chart developed by Kwon et al. [61]. This procedure has already been validated on amblyopic subjects [61, 62]. Five letters spatially filtered to a peak spatial frequency of 2 c/d were presented at various contrasts to the left eye and 5 different letters with complementary contrasts to the right eye at the same spatial locations. Therefore, when viewed binocularly, the letters appeared superimposed. The subject had to report the five most visible letters for 10 trials (Figure 2(b)). The relative contrast of the letters seen by each eye was adjusted by an adaptive method [61, 62] in order to determine the interocular balance point for contrast sensitivity. The interocular balance point is expressed as the ratio in dB between the amblyopic and nonamblyopic eye, so a negative value means that the nonamblyopic eye is stronger, a value close to 0 means that the eyes are well-balanced, and a positive value would indicate that the amblyopic eye is stronger. This test was performed on the same equipment as the movie viewing.

#### 2.4.4. Stereosensitivity

Disparity thresholds were measured using the TNO test (Netherlands Organisation for Applied Scientific Research, distributed by Lameris Ootech BV). It is a duochrome test without monocular clue, based on the principles of Julesz's tests [63], allowing the measurement of stereoscopic acuity from 480 to 15 seconds of arc.

## 3. Results

**Figure 3:**
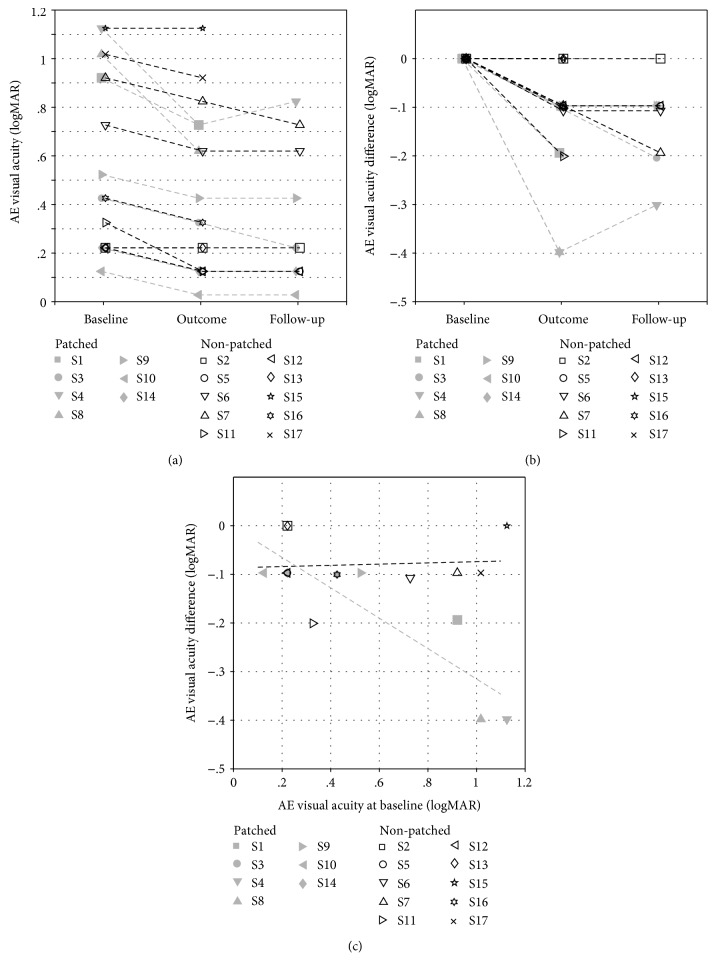
Visual acuity improvement. (a) Visual acuity of the amblyopic eye (AE) of the participants reported at baseline, at the outcome of the training, and at the follow-up control one month later. (b) Visual acuity difference from the baseline of the amblyopic eye. (c) Visual acuity difference from baseline as a function of the initial acuity of the amblyopic eye. Participants from the patched group are indicated with filled grey symbols, and participants of the nonpatched group with open black symbols. Dashed lines represent linear regressions.

We trained 17 subjects, distributed in patched and nonpatched groups (see Materials and Methods) following our protocol to assess the improvement of visual acuity (VA). For most subjects, the VA of the amblyopic eye (AE) improved (lower value in logMAR) at the completion of training compared to before training (the baseline) (see Figure 3(a)). For the nonpatched group (open black symbols), the average value at baseline was 0.54 ± 0.37 logMAR and 0.46 ± 0.38 logMAR at the completion of training. This is a significant improvement of 0.08 logMAR (one-sided Wilcoxon signed rank test, *p* < 0.01), which is equivalent to almost one line on the visual acuity chart. For the patched group (filled grey symbols), the average visual acuity of the amblyopic eye was 0.62 ± 0.40 logMAR at baseline and 0.43 ± 0.28 logMAR at the end of training, resulting in an average improvement of 0.19 logMAR, equivalent to almost two lines on the chart. This improvement is also significant (one-sided Wilcoxon signed rank test, *p* < 0.01) and remained significant at the one-month follow-up (one-sided Wilcoxon signed rank test, *p* < 0.05) whereas it did not quite reach significance for the nonpatched group. To better appreciate these improvements, the difference in VA from baseline is reported in Figure 3(b). The improvement in visual acuity of the amblyopic eye of the participants in the patched group is slightly greater, although this difference is not significant (two-sided Wilcoxon rank sum test, *p* = 0.06) and is more long lasting (because it is still significant at the one month follow-up, whereas it is not for the nonpatched group). In both groups, the training did not affect the VA of the nonamblyopic eye with an average improvement of 0.04 and 0.05 logMAR in the patched and nonpatched groups, respectively (two-sided Wilcoxon signed rank test, *p* = 0.31 and *p* = 0.06, respectively), thus verifying that the improvement was not induced by any learning of the visual acuity measurement itself.

In order to test whether the amplitude of the effect we observe depends on the severity of amblyopia, we plot in Figure 3(c) the difference in VA from baseline as a function of the initial acuity of the amblyopic eye. There is no link between the degree of improvement and the initial severity of amblyopia in the nonpatched group (coefficient of determination *r*^2^ = 0.005, *p* = 0.84). However, there is a significant correlation between the degree of improvement and the acuity of the AE at baseline in the patched group (*r*^2^ = 0.78, *p* < 0.01). The effect is such that in this group, the stronger the amblyopia, the greater the improvement. We did not observe a significant correlation between the amplitude of the effect and the age of the participants in either group (*r*^2^ = 0.003, *p* = 0.87 in the nonpatched group and *r*^2^ = 0.001, *p* = 0.94 in the patched group).

**Figure 4:**
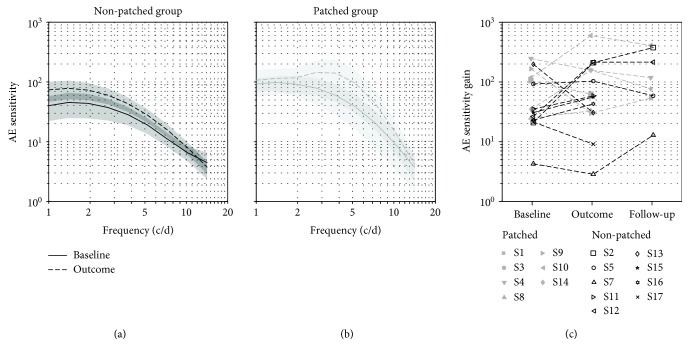
Contrast sensitivity improvement. (a) Contrast sensitivity of the amblyopic eye as a function of spatial frequency at baseline (solid line) and at the training outcome (dashed line) for the nonpatched group. Grey areas represent ±standard error. (b) Contrast sensitivity of the amblyopic eye as a function of spatial frequency at baseline and at the training outcome for the patched group. Same line style as (a). (c) Individual sensitivity gain of the participants at baseline, at the outcome of the training, and at the follow-up control one month later. Participants from the patched group are indicated with filled grey symbols, and participants of the nonpatched group with open black symbols.

Another monocular function we tested was contrast sensitivity. We measured the average contrast sensitivity of the amblyopic eye as a function of spatial frequency before and after training (Figure 4). For the nonpatched group (Figure 4)(a), the contrast sensitivity function at baseline peaks at approximately 1.5 c/d with an amplitude of 45 (solid line) which is in line with previous reports [58, 59]. At the completion of training, the amplitude reaches 78. For the patched group (Figure 4(b)), the contrast sensitivity function at baseline peaks at approximately 1.5 c/d with an amplitude of 84 (solid line). After training, the amplitude reached 134 with a peak shifted to higher frequencies at 3 c/d. In order to test the significance of these improvements, we reported the gain parameter of the sensitivity function as estimated by the qCSF method [57] for each participant at baseline, at the completion of training, and at the follow-up control, after training had been completed in Figure 4(c). This training improvement is not significant for either the nonpatched or the patched group (one-sided Wilcoxon signed rank test, *p* = 0.21 and *p* = 0.34, respectively). It is not different between the two groups either (two-sided Wilcoxon rank sum test, *p* = 0.58). And there is no significant correlation between the amplitude of the improvement and the gain at baseline in either group (respective *r*^2^ = 0.46, *p* = 0.10 and *r*^2^ = 0.10, *p* = 0.41 in the patched and nonpatched group).

**Figure 5:**
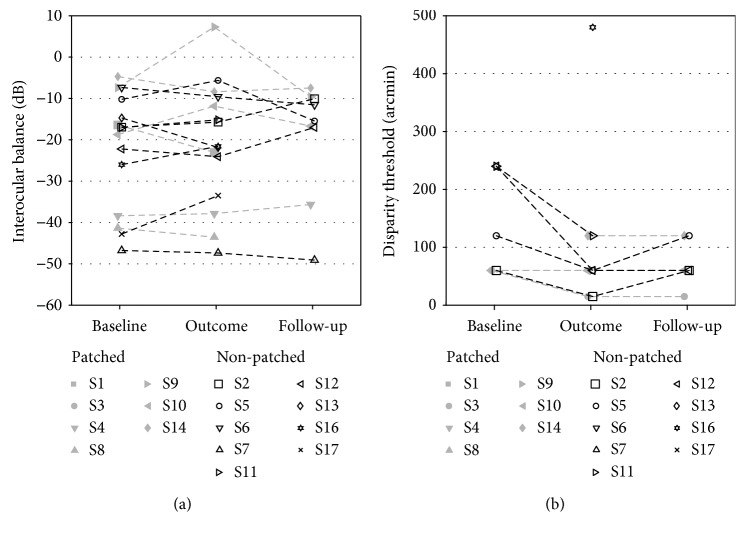
Effect of training on binocular vision. (a) Interocular balance amblyopic/nonamblyopic eye expressed in dB at baseline, at the outcome, and at the follow-up of the training. (b) Disparity sensitivity threshold at baseline, at the outcome, and at the follow-up of the training. Participants from the patched group are indicated with filled grey symbols, and participants of the nonpatched group with open black symbols.

Finally, we tested the effect of the training on two binocular functions: the interocular balance and the stereosensitivity (Figure 5). The interocular balance expressed as the ratio in dB between the amblyopic and nonamblyopic eye is reported for each participant in Figure 5(a). The averages of the balance over the nonpatched group at baseline and at the completion of training are, respectively, −22.67 ± 13.76 and −21.61 ± 12.68 dB. The fact that the value gets closer to zero indicates a small improvement in the balance, although it is not significant (one-sided Wilcoxon signed rank test, *p* = 0.71). For the patched group, a better improvement from −21.16 ± 15.44 to −19.57 ± 19.12 dB was observed; however, this is not significant either (one-sided Wilcoxon signed rank test, *p* = 0.66). Even merging, the two groups, this improvement remained not significant (one-sided Wilcoxon signed rank test, *p* = 0.68).

For stereosensitivity, among the subjects who initially had stereovision, in the nonpatched group, their average stereo threshold improved from 165 ± 90 arcmin at baseline to 64 ± 43 arcmin at the completion of training (Figure 5(b)). However, this improvement was not significant because only four subjects could initially perform the test (one-sided Wilcoxon signed rank test, *p* = 0.06). Additionally, one subject (S16) who previously was not able to perform the TNO test showed a measurable stereosensitivity after training (480 arcmin). In the patched group, only two subjects had a measurable stereosensitivity at baseline. Their average stereo threshold improved from 60 ± 0 arcmin to 38 ± 32 arcmin, but again, this improvement was not significant due to the small sample size (one-sided Wilcoxon signed rank test, *p* = 0.5). Here again, one subject (S14) who was not able to perform the TNO test initially showed a measurable stereosensitivity after training (120 arcmin).

Since there was no statistically significant difference in any measure between the patched and nonpatched groups and since they both were subjected to the same passive dichoptic movie treatment, in order to get more statistical power, we combined the results from the two groups to address the question of whether the treatment per se leads to improvements in visual function in older children and adults with amblyopia. Statistically significant improvements were found in both visual acuity (average improvement from 0.58 to 0.45 logMAR: one-sided Wilcoxon signed rank test, *p* < 0.001) and stereopsis (130 ± 88 arcmin to 55 ± 39 arcmin: one-sided Wilcoxon signed rank test, *p* = 0.03). This is the first report of the successful application of this passive approach in amblyopic older children and adults which complements a previous report of its success in younger amblyopic children [36].

## 4. Discussion

The primary objective of our study was to evaluate the effect of binocular training with passive dichoptic movie viewing on subjects with a stable resistant amblyopia. The training intervention was very minimal compared with classical patching therapy: 9 hours compared with many months. Our results showed that even very short dichoptic movie viewing significantly improved visual acuity of about one line after approximately 9 hours of training over a two-week period; the maximum visual acuity improvement measured was of 3 lines. This improvement is consistent with the results of Bao et al. [25] on teenagers and adults using an altered reality system and Li et al. [36] and Mezad-Koursh et al. [39] on children using passive movie viewing. The visual acuity improvements we observe are comparable with those of Bao et al. (0.08 logMAR improvement in both studies). Although not unexpectedly, they are lower compared to those obtained in children (0.20 logMAR for Li et al. and 0.26 logMAR for Mezad-Koursh et al.). This difference may be explained by the fact that subjects in these studies were children whereas in our study, they were mostly adults, hence showing less plasticity [64].

We also observed an improvement in the monocular peak contrast sensitivity function amplitude, but it was not significant due to the small sample size [25]. Despite the subjects' ability to appreciate the full picture of the movie while we increased the contrast seen by the fellow eye by 10% for each session, the interocular contrast sensitivity balance remained quite stable after training. Bossi et al. [40] and Li et al. [36] observed similar results, contrary to Hess et al. [26], Li et al. [28], and Kelly et al. [37] who observed a reweighting of this balance proportional to the visual gain. The reason for this is unclear; each of the above studies used a different test for binocular balance. The method used in the study by Kelly et al. [37] is similar to that used in the present study; however, they studied children and we studied adults. It may be possible to improve acuity in the absence of any change in binocular function [36, 40].

Most subjects who had measurable stereoscopic vision with the TNO test at inclusion showed an improvement of it although this was not significant due to the small sample of subjects and the fact that disparities larger than 480 arc seconds could not be measured with the TNO test. Indeed, among the 10 subjects of the nonpatched group, only 4 of them had measurable stereoscopic vision at baseline and all of them improved after training. In this nonpatched group, one patient without measurable stereoscopic vision with the TNO test at baseline exhibited measurable stereopsis after training. Only 2 subjects had measurable stereoscopic vision at baseline in the patched group. One improved and one remained constant after training. In this group, one patient without measurable stereoscopic vision at baseline exhibited measurable stereopsis at final evaluation too. The trend is for stereopsis to improve although owing to limitations in our stereo test [65–68] and the reduced stereopsis of our patients. When the results of the two groups were combined, this improvement became statistically significant (130 ± 88 arcmin to 55 ± 39 arcmin: one-sided Wilcoxon signed rank test, *p* = 0.03). This is the first evidence that passive dichoptic movie training improves stereovision in older children and adults with amblyopia.

Our study shows for the first time that a very short period (9 hours) of passive dichoptic movie viewing can improve visual function in adult subjects presenting with a stable and resistant amblyopia. Previously, a similar improvement was shown for an altered reality system in teenagers and adults [25]. One interest of dichoptic movie viewing is its potential to increase compliance in comparison to patching or to other forms of dichoptic training [25, 31, 35, 41, 45, 69, 70]. First of all, because dichoptic movie viewing is passive, it does not require any active participation of the subject, unlike perceptual learning or dichoptic video game play. This is a crucial advantage especially for older subjects who do not want to play video games or even for younger children who may not have the necessary cognitive capabilities. Furthermore, dichoptic movie viewing is very flexible in that it can be used at home and can be adapted to any video content such as virtual or augmented reality approaches [25, 41, 71–74].

The secondary objective of our study was to evaluate if the mechanisms involved in short-term monocular occlusion and dichoptic movie training could be complementary and synergistic and, if combined together, result in a larger therapeutic effect. Short-term monocular deprivation might activate binocular brain plasticity mechanisms via changes in the excitatory/inhibitory balance [48–50, 52, 75, 76] and that could enhance dichoptic training-based improvements.

We observed a trend that such prior monocular occlusion could enhance the effect of training on visual acuity: our results showed a larger improvement of visual acuity in the patched group (0.19 logMAR, almost 2 lines, maximum gain in this subgroup: 4 lines), in comparison to the nonpatched group (0.08 logMAR, almost 1 line, maximum gain in this subgroup: 2 lines); however, the difference was not significant for our sample size.

Two recent studies examined the effect of intermittent monocular patching of the amblyopic eye 2 h per day as a treatment for amblyopia with procedures comparable to our patching [77, 78]. The Lunghi et al. study also involved physical exercise, and the Zhou et al. study involved more patching sessions. They, respectively, reported improvements of 0.15 and 0.13 logMAR in the acuity of the amblyopic eye which is less than the 0.19 logMAR improvement we observed with the combined patching and dichoptic movie viewing procedure.

We do not observe any correlation between the acuity of the amblyopic eye at baseline and the improvement in the nonpatched group (Figure 3(c)). This could indicate that the dichoptic movie training effect in itself does not depend on the strength of amblyopia and that the difference we observe in the improvement between the two groups is not due to their initial acuity differences. Lunghi et al. [77] do not report such correlation either in their patching combined with exercise study. However, we observe a correlation in the patched group such that the stronger the amblyopia, the greater the improvement. This would indicate that the preliminary patching mostly affects severe cases of amblyopia. One explanation could be that the improvement reaches a saturation level in mild cases whereas the combined patching and dichoptic training method would be the only one powerful enough to show a greater improvement in severe cases.

This trend should be investigated with a much larger sample size and possibly a crossover design because there is a good reason to think that these two approaches (ocular dominance plasticity and dichoptic training) may, because of their different dynamics, be mutually beneficial. Preliminary monocular patching might act on short-term adaptation by altering the inhibitory/excitatory balance allowing a rapid change in contrast gain [48–50, 52–54]. On the other hand, dichoptic movie training follows a slower course, probably by involving binocular mechanisms similar to perceptual learning [25, 79] resulting in the longer term establishment of new synaptic connections [55, 56, 80–82]. Thus, there is every reason to think that the change in the excitatory/inhibitory balance may accelerate and/or amplify the plasticity effect induced by the dichoptic training by inducing a more plastic state in the brain before each training session.

There were trends that did not reach significance between either groups for other visual functions: monocular contrast sensitivity, interocular contrast balance, and stereoscopic vision. The results in each group should be considered independently because the two groups were not homogeneous. Indeed, randomization was not possible because of logistic issues (i.e., preliminary patching was not possible for subjects who were coming to the hospital by car or who were coming very early in the morning). In both groups, the subjects can be considered as their own controls because training did not affect the VA of the nonamblyopic eye; this rules out any hypothesis based on the fact that the improvement could have been a consequence of task learning. Furthermore, all participants were used to watching screens (TV or computer) at least one hour a day (average 3.8 hours a day, see Table 1). Hence, adding 1.5 hour of TV watching every 2-3 days did not drastically change their exposure to digital screens, and so it is very unlikely that the improvement we observe could be solely due to the increased time of screen exposure per se.

Apart from these inconveniences, preliminary monocular patching did not really decrease compliance (qualitative report) to the training because it was the amblyopic eye that was patched [77, 78]; hence, it was much less disabling than patching the fellow eye, and the patching was for a much shorter duration compared to what the subjects were used to.

Our training method shows promising results and could be used to power larger scale *randomized controlled trials* to validate this type of treatment. These results were obtained in only six sessions over a 2-week period of training. There are a number of recommendations: extend the training to a longer period than 2 weeks, develop a better measure of stereopsis in the coarse disparity range, one that can provide an individual variability measure for better statistical evaluation, produce a more sensitive test of binocular balance, and extend the periods of monocular occlusion to see if its benefits for dichoptic training can be enhanced.

## Figures and Tables

**Table 1:** Characteristics of amblyopic subjects.

#id	Sex	Age	Group	Type of amblyopia	History of occlusion therapy	Hours/day watching a screen	AE BCVA	TNO (arcsec)	Number of training sessions	Total time of visioning	Follow-up (in days)
1	M	19	P	Mixed	No	3	0.92	NA	6	8.45	NA
2	F	11	NP	Anisometropia	No	2	0.22	60	6	9.12	48
3	F	13	P	Anisometropia	Yes	2	0.42	60	6	9.12	32
4	M	42	P	Strabismus	Yes	5	1.12	NA	6	8.78	40
5	M	47	NP	Anisometropia	Yes	2.5	0.22	120	6	8.38	35
6	M	21	NP	Anisometropia	No	6	0.72	NA	6	9.86	34
7	M	45	NP	Anisometropia	Yes	2	0.92	NA	6	7.55	38
8	F	32	P	Anisometropia	No	2	1.01	NA	6	8.7	NA
9	F	67	P	Strabismus	No	4	0.52	NA	6	8.96	42
10	M	35	NP	Anisometropia	No	15	0.12	60	6	8.8	51
11	M	10	NP	Mixed	Yes	1	0.32	240	6	9.06	NA
12	F	47	NP	Anisometropia	Yes	4	0.22	240	6	8.81	33
13	F	9	NP	Anisometropia	Yes	NA	0.22	NA	6	8.8	NA
14	M	38	P	Anisometropia	No	2.5	0.22	NA	6	10.11	42
15	M	62	NP	Mixed	No	3	1.12	NA	6	10.46	NA
16	F	46	NP	Anisometropia	No	2.5	0.42	NA	6	8.45	NA
17	M	42	NP	Mixed	No	5	1.01	NA	4	7.71	NA

Group assignment: P = patched, NP = nonpatched.

## Data Availability

The data used to support the findings of this study are available from the corresponding author upon request.

## Supplementary Materials

Supplementary 1 Supplementary Movie 1: example movie with 100% contrast images presented to the two eyes. Source video: Lauren Sauvan, wikimedia commons/CC-0.

Supplementary 2 Supplementary Movie 2: example movie with 100% contrast image presented to the left eye and 40% contrast image presented to the right eye. Source video: Lauren Sauvan, wikimedia commons/CC-0.
